# 3D digital exoscope is safe tool in the surgery of olfactory groove meningiomas

**DOI:** 10.1007/s00701-026-06822-6

**Published:** 2026-03-06

**Authors:** Lilli Tolppola, Anni Pohjola, Ville Vasankari, Mika Niemelä, Martin Lehecka

**Affiliations:** Department of Neurosurgery, Helsinki University Hospital and University of Helsinki, P.O. Box 266, 00029 Helsinki, Finland

**Keywords:** Exoscope, Meningioma, Microscope, Neurosurgery, Resection

## Abstract

**Background:**

Digital 3D exoscopes have been introduced as alternatives for operating microscopes in microneurosurgery. It has been hypothesized that exoscope may provide benefits especially at the most anterior skull base, where surgical trajectories often require heavy tilting of the magnification device. We evaluated the safety and practicality of the digital 3D exoscopes in surgery of olfactory groove meningiomas (OGM) during the transition from using a microscope to an exoscope.

**Methods:**

In this retrospective cohort study, we included all consecutive adult patients who underwent surgery for OGM (*n* = 22) by a single senior neurosurgeon between 2016 and 2024 either with a microscope (*n* = 13) or an exoscope (*n* = 9). We reviewed the pre- and postoperative MRIs, patient records (including Modified Rankin Scale (mRS)), and surgical videos of all the patients. The surgical approach was unilateral fronto-temporal in all the cases.

**Results:**

The patients in the exoscope group had larger tumors (median 61cm^3^ (IQR 49) vs. 17cm^3^ (IQR 32)), more clinical symptoms and required more help in their daily activities preoperatively (mRS ≥ 3: *n* = 3 (33%) vs *n* = 1 (8%)). Gross-total or near total resection was achieved in all the patients. The exoscopic surgeries took longer (165 min (IQR 106) vs. 121 min (IQR 27)), probably due to the larger tumor sizes. Two severe complications occurred, one in each group (post-op hematoma and blindness of ipsilateral eye). Clinical outcomes were nearly the same in both groups. At the 3-month follow-up, eight (89%, exoscope) and 12 (92%, microscope) patients were independent (mRS 0–2). Horizontal adjustments were more common when operating with the microscope (median 251 (range 148–359) vs. 103 (range 19–187)) while tilting movements were more frequent with the exoscope (median 122 (range 74–182) vs. 76 (range 44–133)).

**Conclusion:**

The surgical outcomes for OGMs remained consistent during the transition from using a microscope to an exoscope. The exoscope is a safe tool in the surgery of OGMs, even when operating on large tumors. The wider range of angular movement of the camera head is particularly advantageous when accessing the anterior skull base. In line with this, exoscope-assisted surgeries relied more on tilting movements, whereas microscope-assisted surgeries required more horizontal adjustment.

**Supplementary Information:**

The online version contains supplementary material available at 10.1007/s00701-026-06822-6.

## Introduction

Olfactory groove meningiomas (OGMs) are usually benign (WHO Grade 1) tumors [17], and account for 8–13% of intracranial meningiomas [18]. Due to their location at the base of the anterior cranial fossa, OGMs often reach considerable size before detection, making surgery often the primary treatment. Preoperative symptoms typically include anosmia, and in the case of larger tumors, neuropsychological impairment [8].

Surgical microscope has been the gold standard for intraoperative magnification in microneurosurgery for the last fifty years. Digital 3D exoscopes have recently been introduced as an alternative tool. [3, 10, 23] When compared to microscope, exoscope-assisted surgery has resulted in similar outcomes in both laboratory studies and clinical series of different neurosurgical pathologies (e.g. spinal dural arteriovenous fistulas [1], aneurysms [21], spinal intradural extramedullary tumors [4]). There are only few reports on the use of exoscopes in meningioma surgery in general but no proper series on skull base meningiomas including OGMs. [5, 7, 14, 19]

Our objective was to assess the results of surgery for OGMs during the transition period from traditional surgical microscope to the digital 3D exoscope. We evaluated the safety and practicality of the digital 3D exoscopes in surgery of these lesions by comparing surgical outcomes, complication rates, and intraoperative dynamics in exoscopic and microscopic surgeries. We hypothesized that the results of exoscope-assisted surgery for OGMs are non-inferior to microscopic ones. We also hypothesized that the exoscope offer particular advantages at the most anterior skull base, where optimal surgical trajectories frequently require heavy tilting of the magnification device – a requirement less pronounced in the pathologies examined in earlier studies [1, 4, 5, 21].

## Material and methods

### Patient population and study design

This is a retrospective cohort study of 22 consecutive adult (≥ 18 years) OGM patients operated by a single senior neurosurgeon (MLe) at the Helsinki University Hospital (HUS) between March 2016 and February 2024. We excluded patients with recurrent meningiomas. During the eight-year study period, the transition from microscope to 3D exoscope happened gradually between the years 2018–2022. Of the 22 patients (19 women), 13 (59%) were operated with a microscope during 2016–2022 and nine (41%) with an exoscope during 2018–2024. After the purchase of our department’s first exoscope at the end of 2020, all but one case has been performed with it. The total follow-up time was 54 patient-years, with a median follow-up of 2.4 years per patient (range 0.2–7.1 years). The study was conducted in accordance with the principles of the Declaration of Helsinki. The study was approved by the research review board of the HUS Neurocenter.

### Clinical and radiological data

We collected data from electronic patient records, digital image archives, histopathology reports, and surgical video recordings. Pre- and postoperative magnetic resonance images (MRI) were analyzed by two independent researchers (APo and VVa), and the following radiological parameters were recorded: preoperative maximal tumor diameter (cm) and volume (cm^3^), presence or absence of edema and optic nerve compression; postoperative residual tumor volume (cm^3^), presence or absence of postoperative complications (infarction, postoperative hematoma). The clinical condition of the patient was evaluated preoperatively, and postoperatively at discharge, at the 3-month follow-up and at the latest follow-up visit. The modified Rankin Scale (mRS) was used to evaluate the clinical conditions. [22] In case the patient had died during the follow-up, the relationship to OGM treatment was evaluated based on the death certificate and clinical records.

### Surgical technique

**Fig. 1:**
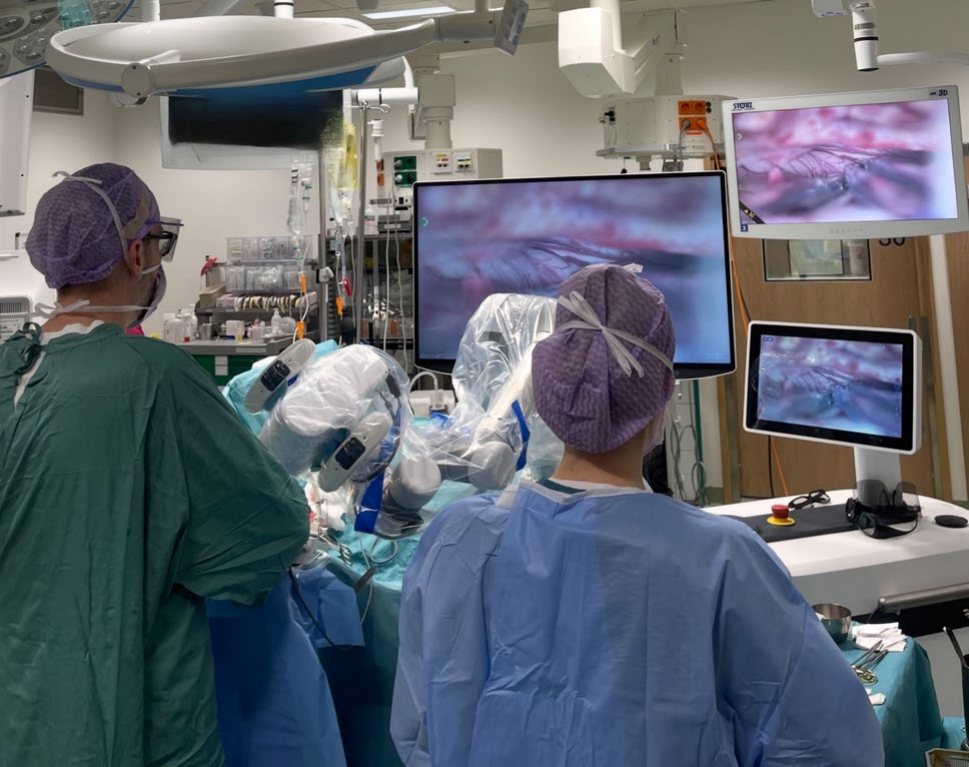
The setup for the exoscope-assisted surgery of olfactorious meningioma with advanced tilting of the camera head

The primary objective of the treatment was to achieve gross total resection (Simpson grade I or II removal) [24] without causing further neurological deterioration. During the operation, effort was made to preserve the olfactory nerves whenever possible. A lateral frontotemporal approach was used in all surgeries (Video 1). Either an operating microscope with a mouthpiece (Pentero 900, ZEISS, Oberkochen, Germany; Kinevo 900, ZEISS, Oberkochen, Germany; and Arveo8, Leica Microsystems, Wetzlar, Germany) or a digital 3D exoscope controlled with a foot pedal (Aeos, Aesculap, B. Braun, Tuttlingen, Germany; or Modus V™, Synaptive Medical, Toronto, Canada) was used as a magnification device (Fig 1). Each surgery was recorded for subsequent video analysis.

Video 1: demonstrates the surgical steps for the removal of an olfactory meningioma using two different magnification devices. © Martin Lehecka, published with permission. Click here to view.

### Surgical outcome

We evaluated the extent of tumor resection using the Simpson grading scale, [24] based on the postoperative MRIs. We recorded the duration of surgery, blood loss, the number of reoperations and possible surgical complications. The complications were classified as severe if they caused permanent neurological deficit or death. All patients underwent contrast-enhanced MRI at the postoperative follow-up visit, typically 2–3 months after the operation. Later follow-ups were usually with 1–3 year intervals depending on the condition of the patient as well as the radiological findings.

### Video analysis

All surgical videos were analyzed by one author (LTo) regarding the following parameters: field of view adjustments, zoom actions, focus adjustments, and video quality. [1] The intradural duration of surgery was measured from the dural incision to the beginning of the dural closure.

The quality of the videos was assessed using the Mean Opinion Score (MOS). [15] The videos were categorized as excellent, good, or fair based on overall qualitative judgment, considering factors such as image blurriness, sharpness, color contrast, and the ability to distinguish between different structures.

### Statistical methods

Descriptive statistics were reported with median, and range or interquartile ranges (IQR). We refrained from multivariable statistical analyses due to limited sample size. We compared categorical variables between the exoscope and microscope groups using Fisher’s exact test and analyzed continuous variables using an independent-sample t-test or Mann–Whitney U test, depending on the normality of the data. We tested normal distribution with the Shapiro–Wilk test and considered a two-sided *p*-value of < 0.05 statistically significant. Due to the small sample size, we did not conduct additional statistical tests (multivariate models). We performed all statistical analyses using SPSS Statistics (version 28.0.0.0 (190)) .

## Results

### Preoperative characteristics

The preoperative characteristics of the study cohort are shown in Table 1. There were some differences between the microscopic and exoscopic groups. The patients in the exoscopic group were younger (median age 57 years (IQR 15) vs. 66 years (IQR 11)) and had larger tumors (median 61cm^3^, IQR 49 vs. 17cm^3^, IQR 32). This resulted in more visual deficits (67% vs. 31%), behavioral/memory problems (78% vs 54%) and need for prior hospitalization (33% vs. 8%) in the exoscope group. Overall, the patients in the exoscope group were more severely affected by their tumors. Interestingly, anosmia was less frequent in the exoscope group (56% vs. 69%) even though the tumors were larger.

**Table 1:** Preoperative characteristics of the 22 patients with OGMs

	Exoscope Group, *n* = 9	Microscope Group, *n* = 13	*P* values
Age [y], median (IQR)	57 (15)	66 (11)	0.19
Female [sex], number of patients (%)	8 (89%)	11 (85%)	1.0
Preoperative residence, number of patients (%)			0.17
Home	5 (67%)	12 (92%)	
Nursing home	1 (11%)	0 (0%)	
Hospital	3 (22%)	1 (8%)	
Preoperative symptoms, number of patients (%)			
Epileptic seizure	2 (22%)	2 (15%)	1.0
Anosmia	5 (56%)	9 (69%)	0.66
Behavioral or memory changes	7 (78%)	7 (54%)	0.38
Gait impairment	2 (22%)	2 (15%)	1.0
Visual field defect	4 (44%)	2 (15%)	0.18
mRS, median (range)	2 (1–5)	1 (1–2)	
mRS categories, number of patients			0.129
0	0	0	
1	1	7	
2	5	5	
3	2	1	
4	0	0	
5	1	0	
6	0	0	
Tumor volume [cm^3^ *], median (IQR)	61 (49)	17 (32)	0.10
Maximum tumor diameter [cm], median (range)	5 (3–8)	4 (3–7)	0.089
Tumors categorized according to their maximal diameter, number of patients (%)			0.11
Small, < 3 cm	1 (12%)	1 (8%)	
Medium, 3–6 cm	4 (44%)	11 (84%)	
Large, > 6 cm	4 (44%)	1 (8%)	
Optic nerve compression [n], number of patients (%)	6 (67%)	4 (31%)	0.19
Peritumoral edema [n], number of patients (%)	8 (89%)	10 (77%)	0.62

### Surgical treatment

**Fig. 2:**
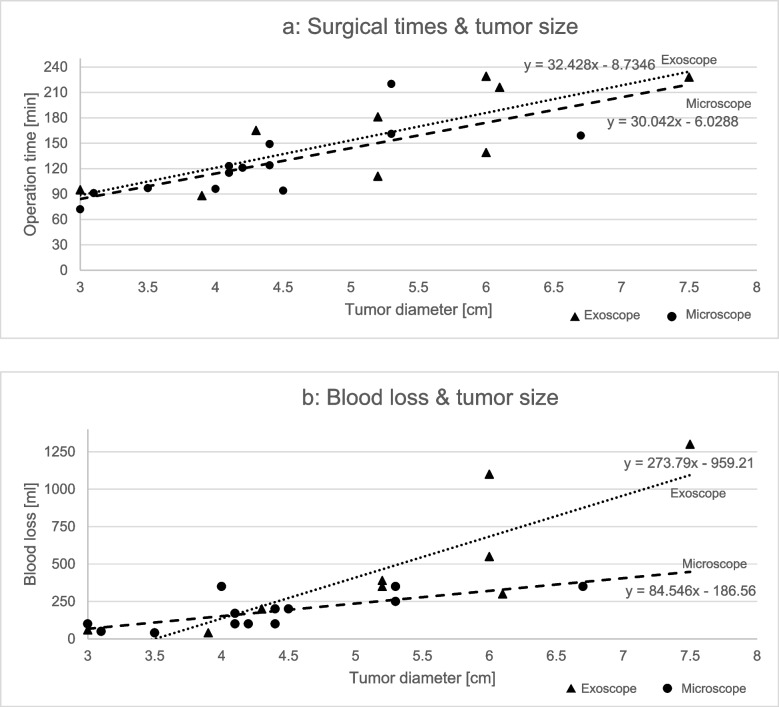
**a** The duration from dural opening to closure compared to tumor size in OGM surgeries **b** Blood loss compared to tumor size in OGM surgeries

The exoscopic surgeries took longer (median intradural surgical time 165 min, IQR 106 vs. 121 min, IQR 27) with more blood loss (median 350 ml, IQR 350 vs. 170 ml, IQR 150) than the microscopic surgeries. These differences were mainly due to tumor size, as it influenced both surgical time and blood loss (Fig. 2a and b). There was no difference in the duration of the exoscopic surgeries over time, as chronologically the first and the second half of the series had similar operation times (mean 154 min, range 88–229 vs. mean 167 min, range 72–162), while the tumor diameter remained similar (mean 5.0 cm, range 9–77 vs. 5.3 cm, range 9–84). Gross- or near total resection was achieved in all the patients. Two patients in the exoscopic and one in the microscopic group had grade 2 meningiomas, all the others were grade 1 tumors (Table 2).

**Table 2:** Surgical parameters

	Exoscope Group, *n* = 9	Microscope Group, *n* = 13	*p* values
GTR + NTR^a^ [n], number of patients (%)	9 (100%)	13 (100%)	
WHO grade [n], number of patients (%)			0.54
1	7 (78%)	12 (92%)	
2	2 (22%)	1 (8%)	
3	0 (0%)	0 (0%)	
MIB1-index [n], number of patients (%)			0.23
0–2%	2 (22%)	7 (54%)	
2–4%	5 (56%)	2 (15%)	
4–6%	0 (0%)	4 (31%)	
> 6%	2 (22%)	0 (0%)	
Blood loss [ml], median (IQR)	350 (350)	170 (150)	0.09
Dural operation time [min^b^], median (IQR)	165 (106)	121 (27)	0.15
Complications [n], number of patients (%)			
Transient hemiparesis	2 (22%)	1 (8%)	0.54
Permanent neurological deficit	0 (0%)	1 (8%)	1.0
Hematoma	1 (11%)	1 (8%)	1.0
Pulmonary embolism	1 (11%)	0 (0%)	0.41
New anosmia	0 (0%)	2 (16%)	0.49

^a)^ Gross- or near total resection

^b)^ Time from the dural incision to the start of dural closure

### Intraoperative video analysis

**Fig. 3:**
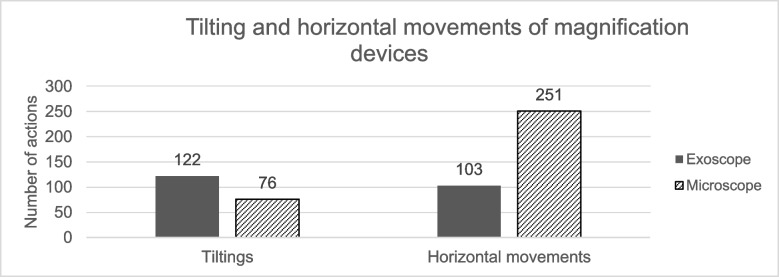
Median number of tilting and horizontal movements of magnification devices in OGM surgeries

Results of the surgical video analysis are shown in Table 3. Horizontal adjustments were more common when operating with the microscope (*p* < 0.001), while tilting movements were more frequent with the exoscope (*p* = 0.004, Fig. 3). Zooming actions were more frequent with microscope (*p* < 0.001). The overall video quality was better with the exoscope in the video recordings (*p* = 0.030). We did not compare the quality of the surgeon’s intraoperative view between the two devices.

**Table 3:** The analysis of the intraoperative videos of exoscope- and microscope-assisted surgery of OGMs

	Exoscope Group, *n* = 9	Microscope Group, *n* = 13	*p* values
All movements^a^ of the magnification device [n], median (range)	225 (105–359)	335 (192–467)	**0.020**
Tilting of the magnification device [n], median (range)	122 (74–182)	76 (44–133)	**0.0040**
Horizontal movements of the device [n], median (range)	103 (19–187)	251 (148–359)	** < 0.0010**
Zooming [n], median (range)			
In	15 (5–25)	35 (8–49)	** < 0.0010**
Out	12 (4–20)	6 (2–12)	**0.020**
Image out of focus [n], median (range)	67 (8–125)	51 (17–115)	0.13
Video quality, n (%)			**0.030**
Fair	0 (0.0%)	2 (15%)	
Good	5 (56%)	10 (77%)	
Excellent	4 (44%)	1 (8%)	

^a)^Tilting movements and movements in plane

### Surgical complications

The surgical complications are shown in Table 2. There were only two severe complications, one in each group. In the exoscope group, one patient with a large meningioma (75 mm) had postoperative hematoma. The hematoma was treated conservatively due to high age, widely metastasized breast carcinoma and severe preoperative disability (preoperative mRS 5). The patient died after ten days. In the microscope group, one patient with a 42 mm tumor lost vision of the right eye. In addition to severe complications, one patient in microscope group suffered a chronic subdural hematoma as a complication which later resolved after burr hole evacuation.

### Immediate clinical and radiological outcome

The postoperative stay at the intensive care unit was similar in both groups (median 1 day, range 1–2; Table 4). Four (44%) patients in the exoscope and two (15%) in the microscope group were transferred for rehabilitation to another hospital, while the rest of the patients were discharged directly to home. Of the six patients requiring rehabilitation, only one was not already hospitalized prior to the surgery (microscope group). For the patients discharged directly to home, the median hospital stay was five days in both groups (exoscope range 3–15 days, microscope range 3–8 days).

**Table 4:** Postoperative course of 22 patients following the surgery of OGM

	Exoscope Group, *n* = 9	Microscope Group, *n* = 13	*p* values
Days in intensive care unit [d], median (IQR)	1 (0)	1 (0)	0.24
Length of hospital stay [d], median (IQR)	5 (6)	5 (3)	0.13
Discharged to home, number of patients (%)	5 (56%)	11 (85%)	0.18
Transferred to a rehabilitation unit, number of patients (%)	4 (44%)	2 (15%)	0.18
mRS at 3-month follow-up visit, median (range)	1 (0–6)	1 (0–3)	0.63
mRS categories, number of patients			
0	2	6	
1	5	5	
2	1	1	
3	0	1	
4	0	0	
5	0	0	
6	1	0	
Residual on postoperative Gd-MRI, number of patients (%)	2 (22%)	4(31%)	1.0
Residual volume [cm^3*^], median (range)	0.6^a^	0.5 (0.4–1.3)	0.35

^a)^ For one patient in the exoscope group postoperative Gd-MRI was not scheduled due to severe memory disorder

There were fewer residual tumors in the exoscope group. Postoperative MRI revealed minor residual tumors (volume ≤ 1.3 cm^3^) in two patients (22%) in the exoscope and four patients (31%) in the microscope group, despite of the larger tumor volumes in the exoscope group. In three cases in the microscope group, intraoperative assessment indicated gross total resection; however, residual contrast enhancement within the operative field was detected in later follow-up MRIs (two in the anterior skull base and on crista galli). In one additional case in the microscope group, tumor remnant was intentionally left due to invasion through the anterior skull base into the ethmoidal cells (patient received postoperative radiation therapy for the remnant). In the exoscope group, both small residual tumors were intentionally left because of tight adherence to the orbitofrontal artery.

### Long-term clinical outcome

At 3-month follow-up, eight (89%) patients in exoscope group and eight (62%) patients in microscope group had better mRS than preoperatively. One patient (11%) in the exoscope group died 10 days after the surgery (post-op hematoma) and one patient (8%) in microscope group had moderate disability. The mRS remained similar at the latest postoperative follow-up.

## Discussion

Supporting our primary hypothesis, the surgery of OGMs with a digital 3D exoscope produced non-inferior results compared to surgeries with a microscope. More tilting movements were observed in exoscope-assisted surgeries, which may be an explanation for the fewer tumor remnants in the exoscope group. There was more blood loss and the surgeries took longer in the exoscope group, but this was probably due to significantly larger tumors. Even though the tumors were larger in the exoscope group, the incidence of severe postoperative complications was low in both groups.

The preoperative tumor size seemed to have the most effect on the course of the treatment. The patients with large tumors had more severe preoperative symptoms, especially neuropsychological problems and visual deficits. This reflected on them requiring more help and hospitalization already prior to the treatment. Intraoperatively there was a clear association between the tumor size and the length of surgery as well as the blood loss. Larger tumors have wider vascular attachment at the skull base as well as more superficial vascular supply from anterior cerebral arteries and its branches resulting in more complicated and time consuming devascularization process during the surgery. The most severe complications also happened to patients with large (> 40 mm) tumors. Our results are in line with previous series regarding tumor size as a predictor for outcome [20] while also reporting similar complication levels between the two groups.

### The maneuverability of the exoscope

Significant differences were observed in the maneuverability of the two magnification devices. Exoscopes allow extreme angulation of the camera head, which can prove particularly advantageous in visualizing the furthest parts of the anterior skull base, often encountered during resection of OGMs. Despite the larger preoperative tumor volumes in the exoscope cohort, less residual tumor was observed in the postoperative MRI. The improved maneuverability of the exoscope appears to facilitate better visualization, potentially leading to more extensive tumor removal. These observations align with previous reports highlighting also the other advantages of exoscopes, including superior image quality, greater ease of repositioning, and expanded magnification range [4, 11, 12, 21]. In this study, we did not compare the intraoperative image quality between the two devices. Furthermore, the higher number of horizontal adjustments required in the microscope group can be explained by the use of a mouthpiece, which allows hands-free horizontal movement of the device. In contrast, tilting the microscope requires removal of at least one hand from the operative field to grab the adjustment handle. The exoscope, however, allows tilting with a foot pedal while preserving uninterrupted bimanual surgical workflown. The microscopic operations were performed during the first half of the study period and the exoscopic surgeries were performed during the second half. The accumulation of surgical skills might have affected the differences in the number of position adjustments between the two devices.

### Exoscopes in intracranial meningiomas

The previous literature on exoscope-assisted surgery for intracranial meningiomas is based only on a few case reports and series with limited sample size [7, 14, 16, 19, 25, 26]. The previous studies have reported enhanced visualization of the surgical field and improved ergonomics for the surgeon in exoscope-assisted surgery [7, 14, 26], while maintaining comparable safety and efficacy to the microscope assisted operations [16, 25]. In this study our findings were similar. There were no differences in the safety and efficacy between the two devices, while superior intraoperative videos were recorded with the exoscope. While this study did not measure ergonomics, our subjective assessment was that the exoscope enables a more ergonomic posture and less strain while working at oblique angles along the anterior skull base (Video 1).

### The benefits and limitations of exoscopes in different steps of OGM surgery

**Fig. 4:**
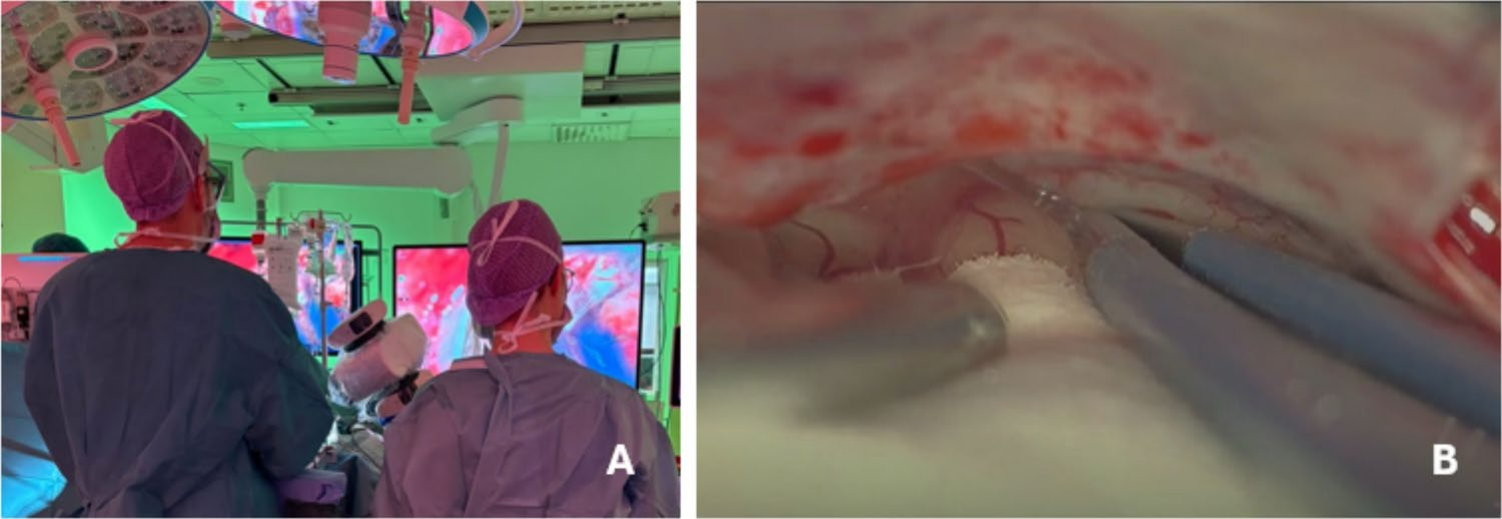
**A**) The surgeon’s position while heavily angulating the camera head during OGM surgery. **B**) Optimal trajectory when approaching the anterior skull base with exoscope

Exoscopes offer several benefits at different stages of OGM surgery. For example, the extreme angulation of exoscope’s camera head allows the surgeon to maintain an ergonomical position while gaining an optimal trajectory towards the anterior skull base, e.g. during frontal lobe retraction or tumor devascularization (Fig. 4A-B). The ability to tilt the camera’s viewing angle without hands allows surgeon to operate continuously during tumor dissection or when performing hemostasis. In more detail, exoscopes offer benefits in a) traversing along the floor of the anterior skull base especially in the region of Crista Galli and the contralateral side from the midline (i.e. heavy lateral tilt) and b) visualization of the most cranial and medial portion of the tumor especially in large tumors (i.e. view towards the surgeon). With operating microscopes these views are the most challenging ones due to mechanical restrictions during frontolateral approaches to the anterior skull base. However, adapting to the use of an exoscope in deep locations, such as the anterior skull base, may require hours of systematic training. It can be challenging to get used to a setting in which the trajectories of the surgeon’s hands and the exoscope’s viewing angle are different from each other.

### Microsurgical removal of OGMs

In the previous studies on microscope-assisted surgery of OGMs, multiple different approaches have been used. The bifrontal approach has been the most common one, resulting in a gross-total resection rate in over 90% of cases [2, 6, 9, 18]. Some authors have reported increased incidence of severe complications (11–26%; e.g. postoperative infarction and hematoma) [2, 6, 13, 18] and higher mortality rate [2, 9, 18] related to the use of bifrontal approach, increasing the interest for using unilateral approaches to access anterior skull base instead. In this study, we only used the lateral frontotemporal approach. Despite of the smaller, less invasive approach and the use of exoscope, gross-total or near total resection was achieved in similar proportion of patients as in prior studies [2, 6, 9, 18, 20] while the incidence of complications was low. This supports the hypothesis that exoscope is a safe tool for the resection of OGMs, even during the adaptation of previous microscope users.

We had a unique opportunity to compare our results with a single surgeon series of operated OGMs published 15 years ago from the same department [20]. Even though the surgeons were different, the patient catchment population, basic treatment concepts and surgical strategy remained similar throughout these years. The present series had fewer patients with small tumors. Postoperative results were similar in both of these series (current vs. previous): anosmia (9% vs 9%), visual deficit (5% vs 8%), postoperative hematoma (9% vs 2%), and deep vein thrombosis (5% vs 3%). Interestingly, there were no patients with postoperative cerebrospinal fluid leaks in the current series, while the rate in the previous series was 9%. This is probably due to a less aggressive strategy of following infiltrative tumor growth through the cribriform plate. The clinical outcomes were also similar with good recovery reached in majority of the patients (73% present vs 65% previous series). Overall, the outcomes of our OGM management have remained consistent over a period of more than 20 years.

### Limitations

This study includes several limitations. Firstly, the retrospective study design inherently carries biases related to data collection and analysis, as it relies on previously recorded information. Secondly, the small sample size restricted the statistical power of the study, preventing the use of multivariate analyses and regression models. Thirdly, the involvement of a single neurosurgeon, who operated on all the patients, ensures consistency in surgical technique and approach, however, limits the generalizability of the results. Fourthly, the focus of the study on a specific patient population within a single hospital setting may not reflect broader clinical practices or outcomes in diverse healthcare environments. Fifth, we did not compare the quality of the surgeon’s intraoperative view between the two devices but analyzed the 2D video recordings postoperatively. Finally, due to the retrospective study design, we were not able to assess the surgeon’s ergonomics. However, we are currently designing a prospective study that will focus on this matter.

## Conclusion

The surgical outcomes for OGMs remained consistent during the transition from using a microscope to an exoscope. The exoscope appears to be a safe tool in the surgery of OGMs, even when operating on large tumors. The advanced tilting of the camera head seems particularly advantageous when accessing the anterior skull base. In line with this, exoscope-assisted surgeries relied more on tilting movements, whereas microscope-assisted surgeries required more horizontal adjustment.

## Supplementary Information

Below is the link to the electronic supplementary material.

Supplementary file1 (MP4 196408 KB)

## Data Availability

The data collected in this study is not openly available.
